# Supervised Learning in All FeFET-Based Spiking Neural Network: Opportunities and Challenges

**DOI:** 10.3389/fnins.2020.00634

**Published:** 2020-06-24

**Authors:** Sourav Dutta, Clemens Schafer, Jorge Gomez, Kai Ni, Siddharth Joshi, Suman Datta

**Affiliations:** ^1^Department of Electrical Engineering, College of Engineering, University of Notre Dame, Notre Dame, IN, United States; ^2^Department of Computer Science and Engineering, College of Engineering, University of Notre Dame, Notre Dame, IN, United States; ^3^Department of Microsystems Engineering, Rochester Institute of Technology, Rochester, NY, United States

**Keywords:** neuromorphic computing, supervised learning, surrogate gradient learning, ferroelectric FET, spiking neural network, spiking neuron, analog synapse

## Abstract

The two possible pathways toward artificial intelligence (AI)—(i) neuroscience-oriented neuromorphic computing [like spiking neural network (SNN)] and (ii) computer science driven machine learning (like deep learning) differ widely in their fundamental formalism and coding schemes (Pei et al., 2019). Deviating from traditional deep learning approach of relying on neuronal models with static nonlinearities, SNNs attempt to capture brain-like features like computation using spikes. This holds the promise of improving the energy efficiency of the computing platforms. In order to achieve a much higher areal and energy efficiency compared to today’s hardware implementation of SNN, we need to go beyond the traditional route of relying on CMOS-based digital or mixed-signal neuronal circuits and segregation of computation and memory under the von Neumann architecture. Recently, ferroelectric field-effect transistors (FeFETs) are being explored as a promising alternative for building neuromorphic hardware by utilizing their non-volatile nature and rich polarization switching dynamics. In this work, we propose an all FeFET-based SNN hardware that allows low-power spike-based information processing and co-localized memory and computing (a.k.a. in-memory computing). We experimentally demonstrate the essential neuronal and synaptic dynamics in a 28 nm high-K metal gate FeFET technology. Furthermore, drawing inspiration from the traditional machine learning approach of optimizing a cost function to adjust the synaptic weights, we implement a surrogate gradient (SG) learning algorithm on our SNN platform that allows us to perform supervised learning on MNIST dataset. As such, we provide a pathway toward building energy-efficient neuromorphic hardware that can support traditional machine learning algorithms. Finally, we undertake synergistic device-algorithm co-design by accounting for the impacts of device-level variation (stochasticity) and limited bit precision of on-chip synaptic weights (available analog states) on the classification accuracy.

## Introduction

Machine learning, especially deep learning has been a *de facto* choice for solving a wide range of real-world complex tasks and has contributed to the unprecedented success story of artificial intelligence (AI) in recent years. Fueled by large datasets and high-performance processors like GPU and TPU, deep learning has exhibited similar or even superior performance compared to human capabilities over a broad spectrum of workloads. However, for applications like smart devices, wearables for healthcare monitoring, or autonomous drones for spatial exploration that require constant real-time information processing, we want to embed implementation of neural networks on the edge. This imposes stringent constraints in terms of power, latency, and footprint area and requires us to rethink the approach toward building hardware for deep learning. Although the architecture of deep neural networks like convolutional neural networks (CNNs) is strongly inspired by the cortical hierarchies, the implementation deviates significantly from the biological counterpart. One obvious point of difference is that neurons are implemented using continuous non-linear functions like sigmoid or ReLu, whereas biological neurons compute using asynchronous spikes that indicate the occurrence of an event. Using such asynchronous event-based information processing may significantly bring down the hardware resources in terms of computational power and footprint area. A recent work established a gain of 54% in area and 45% in power for 65 nm CMOS ASIC implementation of SNN over multi-layer perceptron (MLP) at iso-accuracy and similar architecture (Khacef et al., 2018). Furthermore, with event-based sensors like visual sensors having reached a matured state (Lichtsteiner et al., 2008), SNNs provide a natural choice to be interfaced with them. In the last decade, there has been enormous efforts to build and scale up neuromorphic hardware using CMOS based mixed-signal (Benjamin et al., 2014; Chicca et al., 2014; Park et al., 2014; Qiao et al., 2015) and fully digital (Merolla et al., 2014; Davies et al., 2018) designs. However, there lies several considerations for hardware implementation of SNN that must be undertaken to minimize hardware resources (area and energy), some of which are discussed below.

One major consideration is the choice of the neuronal model and its hardware emulation either in analog or digital domain that will ultimately dictate the compactness and energy efficiency. Biological neurons consist of thin lipid layer membrane whose potential is altered by the arrival of excitatory or inhibitory post-synaptic potentials (PSPs) through the dendrites of the neuron. Upon sufficient stimulation, the neuron generates an action potential and the event is commonly referred to as *firing* or *spiking* of the neuron. To emulate these neuronal dynamics in a hardware, including the transient dynamics as well as the mechanism for neurotransmission, the first ingredient of the implementation is an appropriate choice of the neuron model. Although numerous models have been proposed by drawing inspiration from neuroscience like the biologically plausible complex Hodgkin–Huxley model (Hodgkin and Huxley, 1952) and the Izhikevich model (Izhikevich, 2003), we choose the bio-inspired leaky-integrate-and-fire (LIF) neuron model that provides reduced complexity for hardware implementation while producing the required key dynamics for computation. Spiking LIF neuron can be implemented either in analog or digital domain. While fully digital spiking neurons have been implemented (Merolla et al., 2014; Davies et al., 2018), using analog circuits provides an alternative promising pathway. By using transistors biased in the sub-threshold regime, exponential behaviors can be easily mimicked allowing non-discretized continuous-time neural emulation (Indiveri, 2003; Chicca et al., 2014; Park et al., 2014; Qiao et al., 2015). Recently, Joubert et al. (2012) provided a quantitative comparison between a digital and analog implementation of LIF neuron at 65 nm CMOS technology node with the same level of performance and established an area and energy benefit of 5x and 20x, respectively, for analog over digital design. One pitfall for analog implementation is, however, the usage of large capacitors for emulating the membrane potential. Even with the most drastically scaled technology node, realizing dense on-chip capacitance comparable to biological neuronal membranes (∼10*fF*/μ*m*^2^; Gentet et al., 2000) is challenging. For example, Joubert et al. (2012) implemented the temporal integration property of an analog spiking neuron using a 500 *fF* metal-insulator-metal (MIM) capacitor that requires 100 μ*m*^2^ silicon area while Indiveri et al. (2006) reports using a 432 *fF* capacitance occupying 244 μ*m*^2^ silicon area. Additionally, biological neurons have been shown to be stochastic and this stochasticity adds to the richness of biological computation. With the recent focus on exploiting the physics of functional materials such as ferroelectrics, magnetics, and phase-change materials to build nano-scale devices that can emulate the characteristics of a low-power, stochastic, and capacitor-less spiking neuron, several proposals have been put forward (Sengupta et al., 2016; Tuma et al., 2016; Jerry et al., 2017). In this work, we experimentally demonstrate the essential neuronal dynamics in a 28 nm ferroelectric field-effect transistor (FeFET) technology with ultra-scaled gate length. The membrane potential is represented using the intrinsic ferroelectric polarization and the rich polarization switching dynamics is utilized to perform temporal integration of post-synaptic spikes, thus mimicking an LIF neuron.

The second consideration is the design of synaptic weight storage. Conventional von-Neumann architecture suffers from time and energy spent in moving data between a centralized memory and the processing units. In contrast, a non-von-Neumann architecture allows computation to be done at the location of the stored synaptic weights, thus circumventing the problem of data-movement. Typical examples of such neuromorphic hardware implementing distributed computing include Intel’s Loihi chip with 128 cores each having a local 2 MB static random access memory (SRAM) (Davies et al., 2018) and IBM’s TrueNorth with 4096 neurosynaptic cores each containing 12.75 kB local SRAM (Merolla et al., 2014; Akopyan et al., 2015). Additionally, novel techniques such as time-multiplexing has been proposed to reduce hardware resources or facilitate memory usage efficiently (Akopyan et al., 2015; Davies et al., 2018; Wang et al., 2018; Abderrahmane et al., 2020). Further improvement in energy efficient on-chip training and inference can come from replacing digital SRAM arrays with high density analog synapses that can encode the synaptic weight directly using a physical property of the device such as conductance. Such analog synaptic weight cells can substantially reduce power for both training and inference (Morie and Amemiya, 1994; Burr et al., 2015; Gokmen and Vlasov, 2016). Desirable characteristics of such analog devices include fast and low-power programming of multiple analog states (bit resolution), good retention of the multiple states, and high endurance. Specifically for achieving on-chip training, gradual and symmetric conductance update characteristic is extremely crucial. Recent research efforts have explored numerous potential candidates for building such analog synaptic weight cells including resistive random access memory (RRAM) (Yu et al., 2015; Gao et al., 2015; Prezioso et al., 2015; Wu et al., 2017), phase-change memory (PCM) (Kuzum et al., 2012; Burr et al., 2015; Ambrogio et al., 2018) and FeFETs (Jerry et al., 2018a, 2019; Luo et al., 2019; Sun et al., 2019). In this work, we provide new experimental results of a FeFET-based synaptic weight cell at scaled device dimensions using 28 nm FeFET technology.

Finally, while deep learning, involving non-spiking and often CNNs, has made remarkable progress in achieving human-like performance at solving complex tasks, similar efficient training algorithms have been challenging to design for SNNs. The difficulty in applying traditional deep learning algorithms stems from various factors. First, the notion of time is an important aspect of SNN. As such, a different cost function has to be used that incorporates the notion of time while learning spatiotemporal patterns rather than what’s commonly used in deep learning. Second, spiking neurons are inherently non-differentiable during their time of spike. Over the recent years, several efforts on training SNNs have been undertaken. These include indirect supervised learning like DNN to SNN conversion (O’Connor et al., 2013; Pérez-Carrasco et al., 2013; Diehl et al., 2015; Sengupta et al., 2019), direct supervised learning such as spatiotemporal backpropagation (Wu Y. et al., 2018; Wu J. et al., 2019), and unsupervised training of SNNs using bio-inspired local Hebbian learning rule like spike-time-dependent-plasticity (STDP) (Diehl and Cook, 2015; Panda and Roy, 2016; Kheradpisheh et al., 2018). In this work, we focus on the direct supervised learning scheme. Recently, Zenke and Ganguli (2018) proposed a novel supervised learning algorithm to train multilayer SNNs using a surrogate gradient (SG) based on the membrane potential, known as SuperSpike. In this work, we follow their approach closely by substituting the non-differential derivative of the step-function in the backward pass with a normalized negative part of a fast sigmoid of the membrane potential. Furthermore, we account for the limited bit precision offered by the FeFET-based synapses by considering weight quantization during the training process itself (Wu S. et al., 2018). We quantize the weights in the forward pass while working with high precision gradients. Previous works have shown that DNNs are very well capable of achieving state-of-the-art results with limited precision weights and activations (Choi et al., 2019) as well as quantized errors and gradients (Wu S. et al., 2018). Most modern quantization schemes require additional modification of the learning and inference process by scaling, clipping, or stochastic rounding of variables. Choi et al. (2019), for example, train a separate parameter exclusively for activation clipping and compute a scaling factor for the weights that minimize quantization error. However, note that Choi et al. achieve good results with weights quantized using 2 bits in the forward and backward pass by storing and updating a full precision copy of the weights as well as quantizing them under the consideration of the first and second moment of the weight distribution (SAWB). Since spikes only require 1 bit, the energy consumption in our case is mainly driven by weights. Hence, we focus exclusively on the weight quantization and use the weight quantization method as described by Wu S. et al. (2018) since it imposes marginal quantization overhead. We perform supervised learning on MNIST dataset as an example. We further discuss the impact of FeFET device scaling on the achievable number of analog synaptic weight states (bit resolution) leading to a loss of classification accuracy and potential new avenues for research to circumvent the problem.

## Materials and Methods

### FeFET-Based Analog Adaptive Spiking Neuron

**FIGURE 1:**
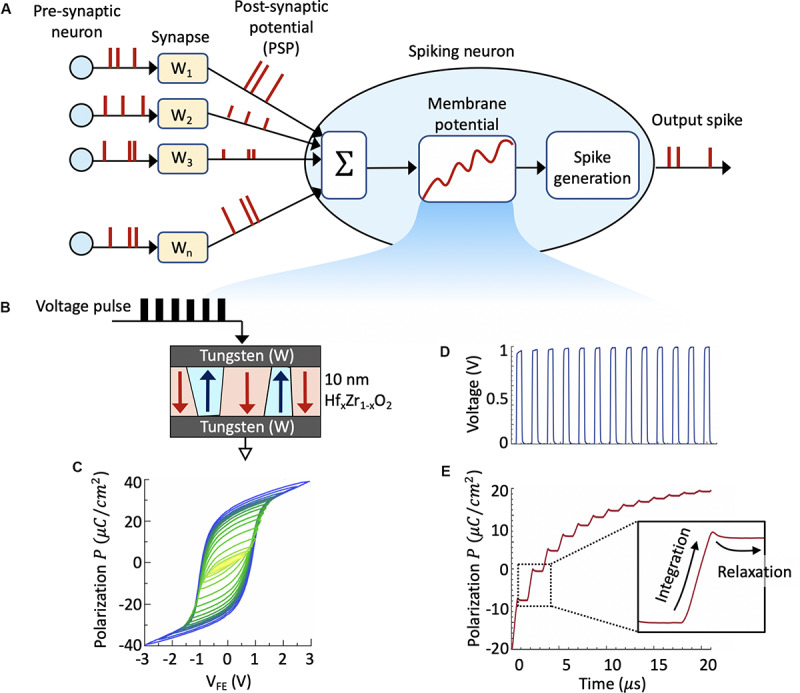
**(A)** Schematic of a spiking neural network consisting of an array of plastic synapses and a spiking neuron. A key element of the spiking neuron is the neuronal membrane, which is represented by the intrinsic state variable, i.e., the ferroelectric polarization. **(B)** Schematic of a metal-ferroelectric-metal (MFM) capacitor consisting of 10 nm ferroelectric Hf_x_Zr_1__–__x_O_2_ thin film sandwiched between two Tungsten (W) metal electrodes that is used to investigating the voltage-dependent polarization switching dynamics. **(C)** Experimentally measured polarization vs electric field (P-E) loop exhibiting saturation as well as minor loops owing to the presence of multiple domains in such ferroelectric thin film. By applying short sub-coercive voltage pulses **(D)**, we can measure the transient polarization switching highlighting the temporal integration of applied voltage pulses and relaxation during the absence of input pulse **(E)**. This inherent polarization dynamics closely resembles the neuronal membrane dynamics of the LIF neuron.

The general working principle of a SNN is as follows. When a synapse receives an action potential, also known as a spike, from its pre-synaptic neuron, it emits a PSP. The PSP in turn stimulates the membrane potential of the post-synaptic neuron. The neuronal membrane potential exhibits temporal evolution where it integrates the PSPs. When the membrane potential crosses a threshold, the post-synaptic neuron fires, i.e., it emits an output spike. Figure 1A illustrates the operation of a simple LIF neuron. Considering a generic LIF neuron, the membrane potential *u* is governed by the following equation:

$$\tau \,\frac{d\,u}{d\,t}=f\,\left(u\right)+\sum w_{i}\,I_{i}$$

where *f*(*u*) is the leak term accounting for the leakage of accumulated charge in the cell membrane, *w*_*i*_ is the synaptic weight, and *I*_*i*_ is the input current that depends on the excitatory or inhibitory PSPs. Upon arrival of excitatory input voltage pulses, the membrane potential continuously evolves in time and as it crosses a threshold, the neuronal circuit sends out an output voltage pulse thereby creating a “*firing event*.” The key idea behind a FeFET-based spiking neuron is to represent the membrane potential *u* by the intrinsic state variable, i.e., ferroelectric polarization instead of the charge stored by a capacitor. As will be discussed next, such dynamics can be achieved within the ferroelectric gate stack of a FeFET that allows realizing compact and low-power spiking neuron.

#### Polarization Switching Dynamics

We start by investigating the voltage-dependent polarization switching dynamics in a 10nm ferroelectric Hf_x_Zr_1__–__x_O_2_ thin film sandwiched between two Tungsten (W) metal electrodes. Such a metal-ferroelectric-metal (MFM) capacitor is illustrated in Figure 1B. The fabricated capacitors have lateral dimensions of 80 μ*m* × 80 μ*m*. Figure 1C shows the experimentally measured polarization vs electric field (P-E) loop exhibiting saturation as well as minor loops owing to the presence of multiple domains in such ferroelectric thin film. Starting from a negative polarization state, where all the dipoles are pointing down, we apply as short voltage pulse. Since the coercive field (V_C_) exhibits a Gaussian distribution in such multidomain thin film, the applied short voltage pulse becomes larger than V_C_ in some of the domains leading to a partial polarization switching in the MFM capacitor.

In order to study the temporal evolution of polarization switching, next we apply short sub-coercive voltage pulses (Figure 1D) and measure the net switching current *I*_*Total*_ as a function of time. The total measured current *I*_*Total*_ will have contribution from two factors—the ferroelectric switching and the linear dielectric response. We subtract the contribution from the dielectric portion to reveal the switching dynamics associated with the polarization alone. Figure 1E shows the transient polarization switching dynamics highlighting the temporal integration of applied voltage pulses and relaxation during the absence of input pulse. The neuronal dynamics is emulated by utilizing the ferroelectric polarization accumulation property (Ni et al., 2018; Saha et al., 2019) that allows temporal integration of PSP. Such ferroelectric polarization switching dynamics bear close resemblance to that of a LIF spiking neuron. It is intriguing to compare the dynamics of the FeFET-based neuron with a standard LIF neuron realized using dielectric capacitor. It is seen that the integration behavior is similar for both the neurons. However, the leak characteristics indicate a surprisingly opposite behavior. As seen in Figure 1E, the transient relaxation in ferroelectric polarization when the voltage pulse is switched off decreases with the increasing in the number of applied pulses. This is contrary to that of a standard LIF neuron built using linear dielectric capacitor, where the discharge rate of the capacitor increases with the pulse number. Such a deviation in polarization relaxation dynamics can be understood by considering the interaction among the ferroelectric domains within the thin film and has been recently studied using phase-field modeling approach (Dutta et al., 2019b; Saha et al., 2019).

#### FeFET Switching Dynamics

**FIGURE 2:**
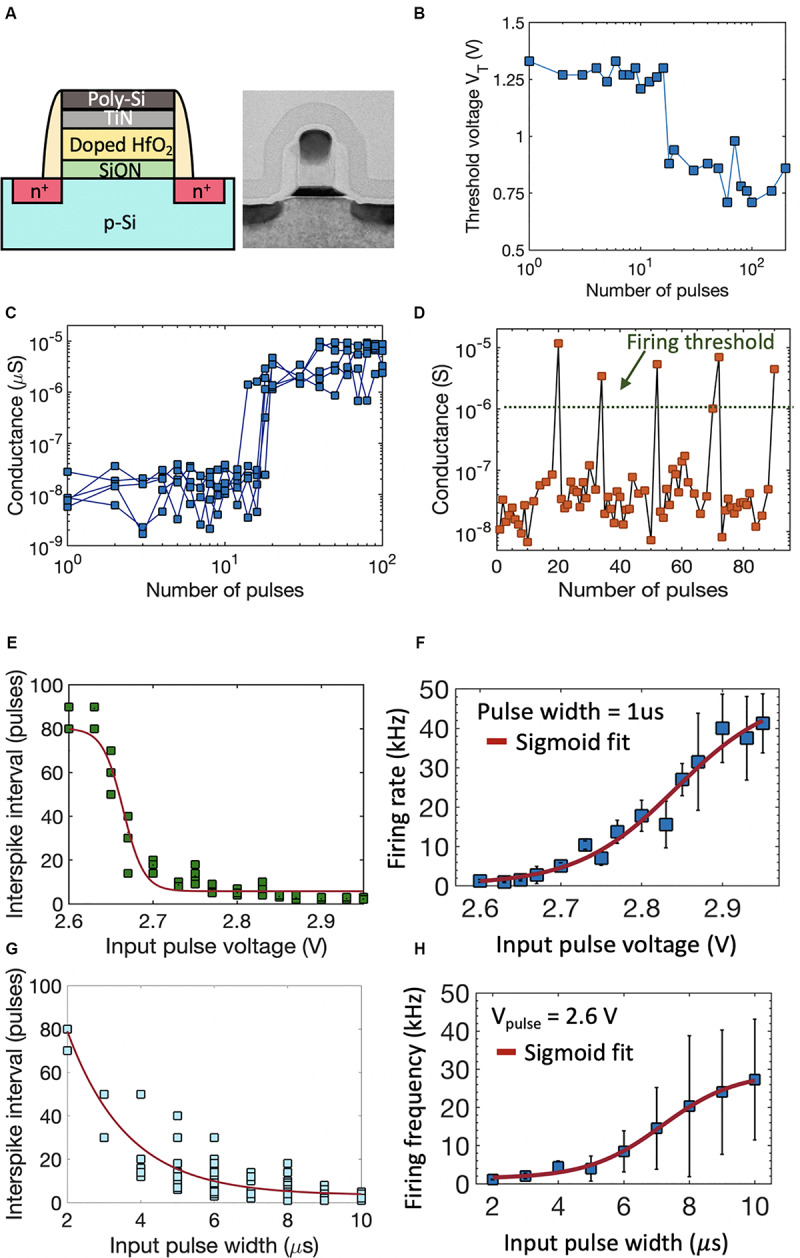
**(A)** Schematic and TEM of a high-K metal gate FeFET with a poly-Si/TiN/Si:HfO2/SiON/p-Si gate stack fabricated at 28 nm technology node. **(B)** On application of successive sub-coercive voltage pulses to the gate of FeFET, the threshold voltage V_T_ gets modulated abruptly from a high-VT to low-VT state. **(C)** Corresponding conductance modulation as a function of number of applied pulses, measured over multiple cycles. **(D)** The integrate-and-fire dynamics of the FeFET neuron. After reaching a conductance threshold, the FeFET is reset to the initial polarization state using a negative gate voltage, which results in a sequence of firing events. **(E,F)** Distribution of inter-spike interval for a range of voltage amplitudes and the corresponding stochastic firing rate of the FeFET neuron. Similar impact of varying the input pulse width on the inter-spike interval and firing rate is seen in **(G,H)**.

Next, we extend the investigation of polarization switching dynamics to FeFETs that consist of a doped-HfO_2_ ferroelectric layer integrated into the gate stack of a conventional MOSFET. Figure 2A shows the schematic and TEM of a high-K metal gate FeFET with a poly-Si/TiN/Si:HfO_2_/SiON/p-Si gate stack fabricated at 28 nm technology node (Trentzsch et al., 2017). All experiments reported here have been performed on FeFETs with channel length of 34 nm and width of 80 nm. On application of successive sub-coercive voltage pulses to the gate of FeFET, the ferroelectric polarization within the Si:HfO_2_ layer switches due to an accumulative effect (Mulaosmanovic et al., 2018a; Ni et al., 2018; Saha et al., 2019), resulting in the modulation of the threshold voltage (V_T_) of the FeFET. As seen in Figure 2B, the V_T_ gets modulated abruptly from a high-V_T_ to low-V_T_ state. The abrupt V_T_ shift arises due to the presence of very few grains (hence ferroelectric domains) within such a scaled device. This in turn causes an abrupt increase in the drain-to-source channel conductance (G_DS_), thereby exhibiting the temporal integration of PSPs in FeFET. Figure 2C shows the measured conductance modulation as a function of the number of applied pulses over multiple cycles. The cycle-to-cycle variation arises from the nucleation dominated ferroelectric polarization switching in FeFET which at the domain level is known to be a stochastic process (Mulaosmanovic et al., 2018b; Dutta et al., 2019a; Ni et al., 2019a). Once G_DS_ exceeds a threshold, the drain current (I_D_) increases and the FeFET is said to “*fire*.” Once in the low-V_T_ state, a negative voltage needs to be applied across the gate and drain/source in order to reset the FeFET to high-V_T_ state. Additionally, since the V_T_ can be gradually increased as well as decreased by applying positive and negative voltage pulses, respectively, this allows the incorporation of both excitatory (*I* > 0) and inhibitory (*I* < 0) inputs without any additional circuitry. Figure 2D shows the continuous conductance modulation due to the application of PSPs and how the integrate-and-fire (IF) dynamics repeats after each reset. Owning to the inherent stochasticity, over multiple IF cycles, a single neuron exhibits a distribution of inter-spike intervals for a range of applied input voltage pulse amplitude or width. Figures 2E,F show the distribution of inter-spike interval for a range of voltage amplitudes and the corresponding stochastic firing rate of the neuron. Similar impact of varying the input pulse width on the inter-spike interval and firing rate is shown in Figures 2G,H. Such stochasticity can be harnessed for emulating the probabilistic activity exhibited by biological neurons (Faisal et al., 2008) without implementing any additional complex circuitry for randomness generation.

#### Implementation of Adaptive Spiking Neuron

**FIGURE 3:**
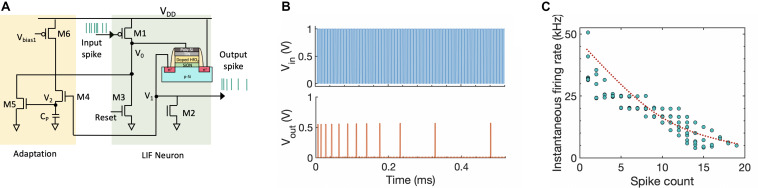
**(A)** Circuit implementation of a FeFET-based spiking neuron. The LIF neuron is implemented using one FeFET and three transistors (M1–M3). Biologically inspired homeostatic plasticity is implemented using additional transistors (M4–M6). **(B)** SPICE circuit simulation of the FeFET-based LIF spiking neuron with spike frequency adaptation. **(C)** Decrease in the instantaneous firing rate of the neuron with each output spike, exhibiting spike frequency adaptation.

We leverage this rich dynamics of the FeFET to implement a low-power spiking neuron circuit consisting of three transistors and one FeFET. Utilizing the temporal integration property of FeFET also allows us to avoid using capacitors for membrane potential, thus providing us an area advantage as well. Figure 3A illustrates the proposed neuron circuit. The input PSPs are applied to the PMOS M1. Initially, both the node voltages V_0_ and V_1_ are at 0 V. As the PSPs are applied, the node voltage V_0_ increases and sub-coercive voltage pulses are applied to the gate of FeFET. Upon application of successive pulses, the FeFET abruptly changes from high-V_T_ to low-V_T_ state and the drain current I_D_ increases. This sends out an output voltage pulse (“*spike*”) as well as increases the node voltage V_1_. Once an output spike is generated, a reset signal is applied to transistor M3. This external reset, initiated by an arbiter, enables array-based operation often seen in large-scale, event-driven, asynchronous systems such as (Indiveri et al., 2011; Benjamin et al., 2014; Park et al., 2014). With M1 being cut-off during the inter-spike intervals, the node voltage V_0_ is pulled down to 0 V. This results in a negative V_GS_ across the FeFET, thus switching the polarization in opposite direction and resetting the FeFET to high-V_T_ state. We also incorporate bio-inspired homeostatic mechanism that regulates the activity of a neuron and lowers the firing rate after every output spike (Liu and Wang, 2001; Benda and Herz, 2003). The homeostatic spike frequency adaptation mechanism is introduced through three additional transistors M4–M6 as shown in Figure 3A. The capacitance C_P_ can be realized by considering the parasitic capacitance of that node. During every output spike event, as the node voltage V_1_ goes high, transistor M4 gets turned on and that in turn increases the node voltage V_2_. The discharge rate of V_2_ can be controlled by adding an additional transistor. As V_2_ increases, M5 gets turned on gradually with every output spike which in turn increases the discharge rate of node voltage V_0_. Thus, the neuron has to integrate over more input PSPs in order to spike which brings down the neuron’s firing rate with every output spiking event, thereby implementing spike frequency adaptation. Figure 3B shows the SPICE circuit simulation of the FeFET-based adaptive spiking neuron. To mimic the stochastic switching dynamics of the FeFET, we introduce a distribution of the coercive field (V_C_) for the ferroelectric domains. We performed Monte Carlo simulation to generate the stochastic spike frequency adaptation as shown in Figure 3C, where the instantaneous firing rate goes down with each output spike. The implication of such a stochastic neuron on the classification accuracy is discussed in section “Results.”

### FeFET-Based Analog Synapse

**FIGURE 4:**
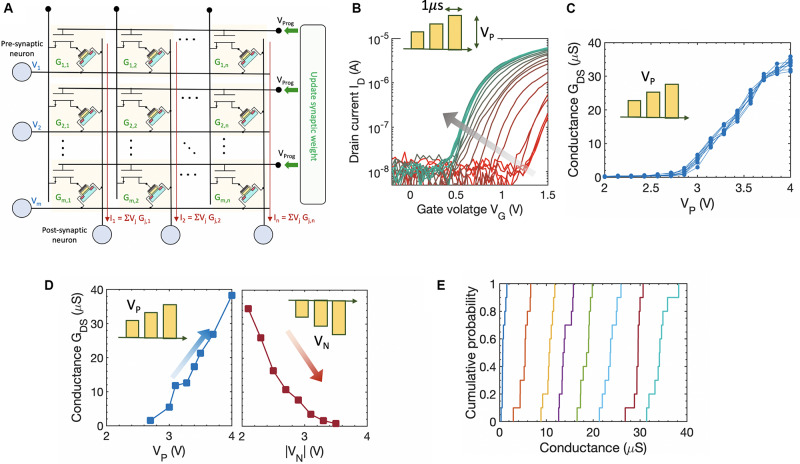
**(A)** FeFET-based pseudo-crossbar array enabling analog vector–matrix multiplication and row-wise parallel weight update of the synaptic weight with column-wise summation. **(B)** Measured Id–Vg characteristics of a 500 nm × 500 nm FeFET upon applying amplitude modulated voltage pulses. Applying progressively increasing gate pulses V_P_ causes the FeFET to transition from the initial high-V_T_ state to lower V_T_ states. **(C)** Corresponding channel conductance of the FeFET upon applying progressively increasing gate pulses V_P_, measured over multiple cycles. **(D)** Measured eight non-overlapping G_DS_ states obtained over multiple cycles using both potentiation and depression pulses that allows the representation of a 3-bit analog weight cell. **(E)** Cumulative distribution of the G_DS_ states corresponding to potentiation pulse scheme obtained over multiple cycles.

The idea of voltage-dependent partial polarization switching in ferroelectric Hf_x_Zr_1__–__x_O_2_ can be leveraged to implement a non-volatile FeFET-based analog synapse. As illustrated in Figure 4A, the FeFET synapse can be integrated into a pseudo-crossbar array that is suitable for row-wise weight update and column-wise summation. Recently, FeFET-based analog synapse has been experimentally demonstrated on 3 μm long and 20 μm wide devices that exhibited 32 non-volatile states (equivalent to 5-bit precision) and a dynamic range of 45x with amplitude modulated programming pulses (Jerry et al., 2018a, b). Here, we provide experimentally measured conductance modulation in a scaled 500 nm × 500 nm high-K metal gate FeFET fabricated at 28 nm technology node (Trentzsch et al., 2017). As shown in Figure 4B, we used the amplitude modulation scheme with pulse widths of 1 μs to modulate the conductance of the FeFET. Applying progressively increasing gate pulses V_P_ causes the FeFET to transition from the initial high-V_T_ state to lower V_T_ states as shown by the I_D_–V_G_ characteristics in Figure 4B. The resulting channel conductance G_DS_ progressively increases as shown in Figure 4C. However, due to the lateral scaling of the device, the number of ferroelectric domains decreases resulting in a reduced number of non-volatile states. Since the typical grain size in 10 nm HfO_2_ is around 10–15 nm, it can be estimated that there will be around 1000 domains for a 500 nm × 500 nm FeFET. This also results in cycle-to-cycle (as well as device-to-device) variation, since the stochastic domain switching contribution from individual domains becomes more pronounced (Ni et al., 2019a). The inherent stochasticity results in a variation of the conductance states measured over multiple cycles for each voltage applied as shown in Figure 4C. We choose eight non-overlapping G_DS_ states obtained over multiple cycles using both potentiation and depression pulses as shown in Figure 4D that allowed the representation of a 3-bit equivalent analog weight cell. Figure 4E shows the cumulative distribution of the G_DS_ states corresponding to potentiation pulse scheme obtained over multiple cycles. This indicates that while FeFETs are a promising candidate for non-volatile analog synapse, the number of available non-volatile states drastically reduce at the scaled node (Dutta et al., 2019a; Ni et al., 2019a). The implications of such a reduced bit-precision on learning algorithms are discussed next. This challenge also opens up new avenues of research both at the material/device/circuit level, as well as at the algorithmic level. For example, a FeFET-based synapse has been recently proposed that utilizes hybrid precision training and inference to overcome the challenge of limited bit precision (Sun et al., 2019).

### Model of FeFET-Based Analog Spiking Neuron and Synapse

The inherent ferroelectric polarization switching dynamics closely resembles the neuronal membrane dynamics of the LIF neuron and can be captured by a modified quasi-LIF neuron model. Our description of the FeFET-based neuron model builds upon the traditional LIF neuron presented by Diehl and Cook (2015) as given below:

$$\frac{d\,v}{d\,t}=\frac{\alpha \,v_{r\,e\,s\,t}-v}{\tau_{l\,e\,a\,k}}+\frac{\sum \left(g_{e}\,\left(E_{e\,x\,c}-v\right)+g_{i}\,\left(E_{i\,n\,h}-v\right)\right)}{\tau_{i\,n\,t\,e\,g\,r\,a\,t\,e}}$$

$$\tau_{g\,e}\,\frac{d\,g_{e}}{d\,t}=-g_{e}$$

$$\tau_{g\,i}\,\frac{d\,g_{i}}{d\,t}=-g_{i}$$

$$\tau_{\alpha }\,\frac{d\,\alpha }{d\,t}=-g_{e}$$

where *v* is the membrane potential, *v*_*rest*_ denotes the resting potential of the neuron, and *E*_*exc*_ and *E*_*inh*_ are the equilibrium potentials of excitatory and inhibitory synapses. τ_*leak*_ and τ_*integrate*_ are the time constants associated with for the leakage and integration phase of the neuron, respectively. When the neuron’s membrane potential crosses the membrane threshold *v*_*thres*_, the neuron fires and the membrane potential is reset to *v*_*reset*_. We incorporate the quasi-leaky behavior (decrease in the leak rate of the neuron during the inter-spike interval) into the neuron model by using a variable resetting voltage by multiplying *v*_*reset*_ with a parameter α that changes with each incoming spike. Additionally, this quasi-leak behavior can also be incorporated into the model by using a variable τ_*leak*_ that also depends on the membrane potential *v* (Dutta et al., 2019b). However, owing to very small relaxation dynamics, one can also ignore the leaky behavior of the FeFET-based neuron and treat it as a perfect IF neuron (Mulaosmanovic et al., 2018a). Furthermore, we use an adaptive threshold regime to regulate the neuron’s activity. Once a neuron hits the threshold and issues a spike, this neuron’s threshold increases by a fixed amount, thereby making it harder for this neuron to spike again and prioritizing activities of other neurons. However, the threshold increase only happens until the neuron threshold reaches a maximum level, after which the threshold is not changed by issued spikes anymore.

Synapse models have been incorporated following Diehl and Cook (2015) where the synaptic conductance changes instantaneously by weight *w* when a presynaptic spike arrives at the synapse, else the conductance decays exponentially. *g*_*e*_ and *g*_*i*_ are the conductances of the excitatory and inhibitory synapse, respectively. τ_*ge*_ and τ_*gi*_ are the time constants of the excitatory and inhibitory PSPs, respectively.

This model is then discretized with a standard Euler method so we can use discrete time steps in our simulation. The discrete time version of the models is expressed as:

$$v\,\left[n+1\right]=\alpha \,E_{r\,e\,s\,t}+\beta \,v\,\left[n\right]+g_{e}\,\left[n\right]\,E_{e\,x\,c}+\beta \,g_{e}\,\left[n\right]\,v\,\left[n\right]+g_{i}\,\left[n\right]\,E_{i\,n\,h}+\beta \,g_{i}\,\left[n\right]\,v\,\left[n\right]$$

$$g_{e}\,\left[n+1\right]=e^{-\frac{\Delta_{t}}{\tau_{g_{e}}}}\,g_{e}\,\left[n\right]$$

$$g_{i}\,\left[n+1\right]=e^{-\frac{\Delta_{t}}{\tau_{g_{i}}}}\,g_{i}\,\left[n\right]$$

$$\alpha \,\left[n+1\right]=e^{-\frac{\Delta_{t}}{\tau_{\alpha }}}\,g_{e}\,\left[n\right]$$

where *v*[*n*] is the discretized membrane potential of the neuron at time step *n*. We use a single membrane time constant τ_*v*_, accounting for both τ_*leak*_ and τ_*integrate*_. $\beta =e^{-\frac{\Delta_{t}}{\tau_{v}}}$ captures the decay in the membrane potential during a Δ_*t*_ time step.

### Supervised Learning for SNNs

The success of deep learning in recent years has largely been attributed to the power of supervised learning techniques and gradient based learning (Lecun et al., 2015). Given an objective function, backpropagation adjusts parameters and weights of a given network so that its objective function is minimized. In DNNs, weights are updated in multiple layers organized hierarchically enabling it to learn complex classification or regression functions. Two critical challenges must be overcome in order for SNNs to gain similar success: (a) The development of hierarchical “deep” networks like (Panda and Roy, 2016; Kheradpisheh et al., 2018) which can learn complex representations and (b) enabling the application of gradient based learning to deep spiking neural networks (SSNs). Several studies have proposed ways to train SSNs in order to address the above challenges, such as (Gütig, 2014; Anwani and Rajendran, 2015). However, these approaches did not sufficiently enhance the representative power of SNNs, nor did they enable the development of deeper more complex networks. Neftci et al. (2019) identified four streams of research attempting to train SNNs with hidden units: (i) biologically inspired local learning rules, (ii) translating conventionally trained “rate-based” neural networks to SNNs, (iii) smoothing the network model to be continuously differentiable, and iv) defining a SG as a continuous relaxation. Fortuitously, the SG-based methods simultaneously address the two challenges presented and form the basis for the remainder of this article. This method allows us to build upon the solid research base of backpropagation with only marginal modifications of the spiking network model.

#### Surrogate Gradient Learning

Historically, the spike function in SNNs prevented the application of gradient based learning rules due to the discontinuities induced by the non-differentiable spikes, consequently “*stopping the gradient from flowing*.” This in turn results in backpropagation failing to function correctly. The SG method substitutes the gradient in the backward pass of the backpropagation with a differentiable proxy or surrogate. This surrogate is generally based on the membrane potential of a neuron. As a result of this new gradient, the non-differentiability is circumvented, and the gradient can propagate. Using this gradient-based update rule, standard solutions to the credit assignment problem can be applied. Thus, given a global loss function, we can apply traditional gradient-based learning methods such as backpropagation through time (BPTT) (Huh and Sejnowski, 2018) or other learning rules such as three factor learning rules (Zenke and Ganguli, 2018) to SNNs. Most modern machine learning libraries (e.g., PyTorch or TensorFlow) provide autograd functionalities which facilitate the gradient computation. Our experiments used BPTT as gradient-based learning method in conjunction with a SG in the backward pass. The SGs were computed by applying the normalized negative part of a fast sigmoid on the membrane potential.

#### Quantization

As highlighted earlier, the on-chip FeFET-based analog synapse provides limited bit precision ranging from 5 to 3 bits in scaled devices. Efficient implementation of (i) off-chip training followed by reduced bit precision for inference mode and (ii) on-chip learning and inference with reduced bit precision, both demand efficient training algorithm taking into consideration the quantization in synaptic weights. To accurately model the effect of quantizing the weights, we follow the procedure outlined in Wu S. et al. (2018). Weights are quantized by restricting them to a feasible range [−1 + σ(*b*_*w*_), +1−σ(*b*_*w*_)], where σ(*b*) = 2^1−*b*^ and *b*_*w*_ is the number of bits encoding the weight. Weights in each layer are further scaled by γ:

$$\gamma =2^{r\,o\,u\,n\,d\,\left[\log_{2}\left(\frac{\left(\frac{1}{\sigma \,\left(b_{w}\right)}-0.5\right).\sigma \,\left(b_{w}\right)}{\sqrt{\frac{3}{f\,a\,n\,i\,n}}}\right)\right]}$$

where *fanin* represents the number of connections into a layer. Weights are also uniformly initialized in their feasible range and clipped to the range after each update during training.

## Results

**FIGURE 5:**
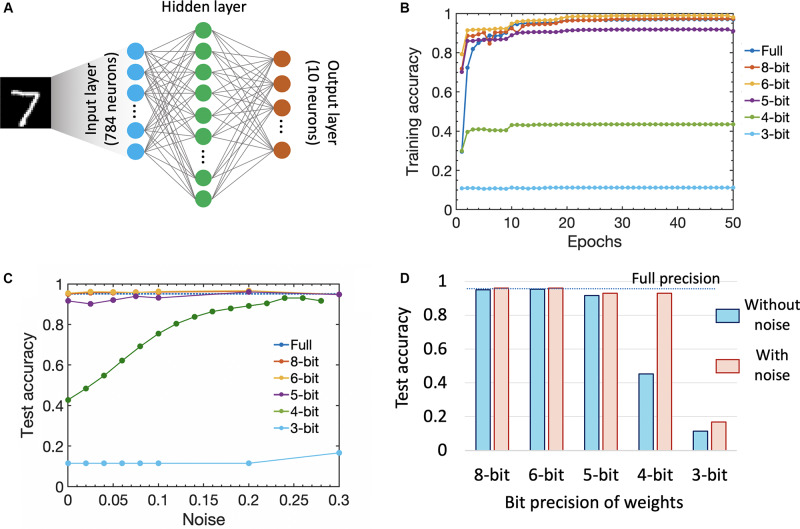
**(A)** We performed supervised learning on MNIST dataset using a three-layer SNN. The input layer consists of 784 neurons while the output layer has 10 neurons to classify the digits. We additionally use a Bayesian hyperparameter optimization approach (Bergstra et al., 2013) to determine the number of neurons in the hidden layer. **(B)** Training accuracy as a function of the number of training epochs for different synaptic weight bit precision (analog states). **(C)** Impact of stochastic noise (incorporated as uniform threshold noise) on the test accuracy for different weight bit precision. **(D)** Comparison of test accuracy for different weight bit precision with and without noise.

We performed supervised learning on MNIST dataset (using the standard train/test split of 60,000/10,000) using a three-layer SNN as shown in Figure 5A. The input layer consists of 784 neurons while the output layer has 10 neurons to classify the digits. The number of hidden layer units is an architectural question which can have significant impact on the performance. We used a Bayesian hyperparameter optimization approach (Bergstra et al., 2013) to determine the number of hidden layer neurons, learning rate, input multiplier (scaling of the input spikes), scaling coefficient for the τ_*leak*_ in relation to τ_*integrate*_, size of the regularizer, batch size, and saturation threshold. All these parameters have an impact on the performance and are sensitive to the dataset of the network. We use the saturation threshold method taken from Yousefzadeh et al. (2018) which prevents the firing threshold from being further increased once it surpasses the saturation threshold, e.g., once it issued a certain number of spikes. For the hyperparameter optimization, we gave the optimizer ranges for the mentioned parameters and programmed it to train a sampled configuration for 12 epochs. Overall, we constrained the optimizer to use 75 evaluations and come up with the best configuration. The number of discrete time steps remained fixed at 80. In our simulations, we used a negative loglikelihood loss function, which performed classification by integrating the last layer’s neurons membrane potential over time and selecting the class of the neuron with the largest integration value.

Since the number of analog states that can be represented by a single FeFET-based synaptic weight cell decreases as we scale the device, it is important to consider the impact of bit precision of the synaptic weights (number of analog states) on training as well as test accuracy. Hence, in our simulation, we varied the weight bit precision from a high value of 8 bits (that would require a single FeFET to represent 256 analog states) to 5 bits (32 analog states demonstrated in a 3 μm × 20 μm device (Jerry et al., 2018a, b) down to 3 bits (eight analog states demonstrated in this work). We also compared the results against the full 32-bit floating point precision available on a CPU. Figure 5B shows the training accuracy as a function of the number of training epochs for various precision of the synaptic weight without introducing any stochastic noise in the simulation. It is seen that while up to 6 bits, we get a test accuracy of 95.4% which is comparable (or even better) to that of the full precision accuracy of 95.1%. The accuracy starts decreasing to 91% for 5 bits and drastically down to 43.5% for 4 bit precision. The increase of accuracy at 6 bits can be seen as the regularizing effect of more coarse weights. The sharp decrease of accuracy for 5 or fewer bits likely indicate the tolerance threshold of SNNs toward reduced weight precision and the lack of information in spikes coupled with weights after a certain weight quantization level. Note that previous works like Choi et al. achieve good results with 2-bit weight quantization in the forward and backward pass by storing and updating a full precision copy of the weights as well as quantizing them under the consideration of the first and second moment of the weight distribution (SAWB). In contrast, our results are obtained with only quantized weights in both the forward and backward pass as well as a linear quantization step reflecting the capabilities of our proposed device.

We further studied the impact of stochastic neurons on the overall performance of the SNN by introducing a uniform noise around the membrane threshold which can be mimicked by the stochastic neuronal dynamics (as shown in Figure 2). Figure 5C shows the impact of noise on the test accuracy. The accuracy for 5–8 bits increased to 96%. Interesting the accuracy for 4-bit weights improved substantially with more noise around the threshold which is in accordance with previous works on ordinary quantized DNNs (Wu S. et al., 2018; Choi et al., 2019). Over a population of neurons and multiple times steps, the threshold with more noise becomes more like a soft function (e.g., sigmoid, softmax, or tanh) rather than a hard threshold and hence more similar to ordinary DNNs which allows for reduced weight precision. In the case of 3-bit weights, we were not able to compensate for the granularity of the weights with noise around the threshold. Figure 5D shows the testing accuracy as a function of various weight precision with and without noise indicating that having a stochastic SNN helps improve the classification accuracy in the presence of reduced weight precision. As mentioned earlier, another way to further improve the accuracy will be to resort to a CMOS-augmented FeFET-based hybrid synapse design that can provide hybrid precision training and inference to overcome the challenge of limited bit precision (Sun et al., 2019).

## Discussion

In this work, we exploit the rich dynamics of ferroelectric polarization switching in FeFET to realize compact and low-power analog spiking neuron and synapse. The membrane potential of the spiking neuron is represented by the intrinsic ferroelectric polarization of the FeFET. The neuronal dynamics is emulated by utilizing the polarization accumulation property (Ni et al., 2018; Saha et al., 2019) that allows temporal integration of PSP. This allows the realization of a capacitor-less analog spiking neuron which proves to be compact and low power. Table 1 shows a comparative study between our FeFET-based analog spiking neuron and various other proposals. Compared to CMOS-based realization of LIF neuron that requires more than 20 transistors and an explicit capacitor, our proposal of FeFET-based spiking neuron requires seven transistors including one FeFET. To estimate the areal requirements, we performed a layout using a 45nm technology node. The estimated area is approximately 1.74 × 1.18 μ*m*^2^ which would be much smaller than the capacitor-based CMOS circuits. For example, Joubert et al. (2012) realized an analog spiking neuron with a footprint area of 120 μ*m*^2^ at 65 nm technology node, of which 100 μ*m*^2^ was dedicated to realizing the 500 *fF* capacitor. Our estimated footprint area in terms of feature size F is at least 4x lower than this. Similarly, Indiveri et al. (2006) report using a 432 *fF* capacitance occupying 244 μ*m*^2^ silicon area. The energy dissipated by our FeFET-based analog neuron is comparable to the analog neuron implementation by Joubert et al. (2012) while it is 4x lower than the digital implementation and 90x lower than the energy dissipated by Indiveri et al. (2006). Compared to PCM-based neuron that requires additional digital circuitry like a latch and a NOR logic gate (Tuma et al., 2016), our FeFET-based neuron dissipates 40x lower power and occupies at least 2.5x lower area in terms of feature size F. Compared to insulator-to-metel phase-transition vanadium dioxide (VO_2_)-based neuron (Jerry et al., 2017), FeFET-based neuron dissipates 300x lower power.

**TABLE 1:** Comparative study between various hardware implementations of spiking neuron.

	Indiveri et al., 2006	Joubert et al., 2012		Tuma et al., 2016	Sengupta et al., 2016	Jerry et al., 2017	This work
Neuron type	LIF	Analog LIF	Digital LIF	LIF	LIF	Piecewise linear FHN	LIF
Material	CMOS	CMOS	CMOS	Phase change (PCM)	Magnetic tunnel junction (MTJ)	Vanadium dioxide (VO_2_)	Ferroelectric HZO
Technology	800 nm	65 nm	65 nm	14 nm	–	–	45 nm
Integration mechanism	Capacitor charging	Capacitor charging	–	Joule heating	Magnetization dynamics	Capacitor charging	Polarization accumulative
Circuit elements	22 Transistor + one capacitor	33 Transistor + one capacitor	Pulse generator, counter, and comparator	One PCM + digital circuit	Two MTJs + four transistors	One VO_2_ + one transistor + one capacitor	One FeFET + six transistors
Stochasticity	Yes	No	No	Yes	Yes	Yes	Yes
Power or energy/spike	900 pJ	2 pJ	41.3 pJ	120 μW	–	11.9 μW	1–10 pJ
Firing rate	200 Hz	2 MHz	2 MHz	35–40 kHz	–	30 kHz	50 kHz
Area	2573 μ*m*^2^	120 μ*m*^2^	538 μ*m*^2^	0.5–1 μ*m*^2^	–	–	2.05 μ*m*^2^

The intrinsic ferroelectric polarization switching mechanism being a stochastic process (Mulaosmanovic et al., 2018b, Mulaosmanovic et al., 2018c; Dutta et al., 2019a; Ni et al., 2019a), the FeFET-based spiking neuron exhibits stochastic firing that maybe useful for building stochastic neural networks like neural sampling machine with novel properties like inherent weight normalization (Detorakis et al., 2019), for applications like modeling uncertainties in neural networks (Gal and Ghahramani, 2016) and for probabilistic inferencing (Pecevski et al., 2011). One key limitation of FeFET-based neuron compared to generalized neuron model utilized in neuroscience and CMOS-based circuits is that the membrane potential is represented by the intrinsic ferroelectric polarization state variable and the associated stochasticity arises directly from the ferroelectric domain nucleation process. Hence, the degree of tuning the neuronal parameters and the stochastic response is limited which might be disadvantageous for algorithms in which the parameters and the stochasticity have to be tightly controlled.

The FeFET-based analog synapse is realized using voltage-dependent partial polarization switching in multi-domain ferroelectric thin film (Jerry et al., 2018a, b). Recent experimental works have shown the ability to program FeFETs with voltage pulse widths as low as 50 ns (Jerry et al., 2018b) while the programming voltage can be brought down from 4 to 1.8 V by engineering the gate stack by adding an additional metal layer between the ferroelectric capacitor and MOS capacitor (Ni et al., 2019b). Table 2 shows a comparative study between FeFET-based analog synapse and various other candidates like PCM (Burr et al., 2010; Athmanathan et al., 2016; Ambrogio et al., 2018) and RRAM (Lee et al., 2012; Wu et al., 2017; Wu W. et al., 2018; Luo et al., 2019). One major benefit of using FeFET for implementing analog synapse is the reduced variability to less that 0.5% (Luo et al., 2019) and an order of magnitude reduction in write energy (Dünkel et al., 2018; Ni et al., 2019b). The cell area is comparable to that of PCM and RRAM. One limitation of FeFET-based analog synapse is the achievable number of non-volatile conductance states as we scale down the device. While a recent experiment on 60 μm^2^ size FeFET devices exhibited 32 conductance states (equivalent to 5-bit precision) (Jerry et al., 2018a, b), in this work, we achieved eight non-overlapping conductance states (equivalent to 3 bits) in a 0.25 μm^2^ size device. The precision of synaptic weight overed can be further improved by resorting to hybrid mechanisms like the recently proposed two transistor-one FEFET (2T1F) hybrid weight cell that allow up to 64 states with improved non-linearity and asymmetry factors (Luo et al., 2019; Sun et al., 2019). Similar hybrid schemes have been applied to other novel devices like the three-transistor, one-capacitor, and two PCM (3T1C+2PCM) weight cell (Ambrogio et al., 2018).

**TABLE 2:** Comparative study between various hardware implementations of analog synaptic weight cell.

	PCM	RRAM	FeFET
Material	GST	TaO_x_/HfO_x_	Hf_x_Zr_1–x_O_2_
States	8	128	8
Variation	∼1.5%	∼3.7%	<0.5%
Write voltage	2.5 V	1.6 V	4 V
Write energy	30 pJ	∼10 pJ	0.1 pJ
Cell area	25F^2^	24F^2^	24F^2^

## Conclusion

In summary, we explore the rich polarization switching dynamics and non-volatile nature of FeFETs and propose an all FeFET-based SNN neuromorphic hardware that can enable low-power spike-based information processing and co-localized memory and computing (a.k.a. in-memory computing). We experimentally demonstrate the essential neuronal and synaptic dynamics in a 28 nm high-K metal gate FeFET technology. Furthermore, we implement a SG learning algorithm on our SNN platform, thus enabling us to perform supervised learning. As such, the work provides a pathway toward building energy-efficient neuromorphic hardware that can support traditional machine learning algorithms. We also undertake synergistic device-algorithm co-design by accounting for the impacts of device-level variation (stochasticity) and limited bit precision of on-chip synaptic weights (available analog states) on the classification accuracy and highlight possible avenues of future work to overcome the current challenges such as resorting to hybrid precision training and inference.

## Data Availability Statement

The datasets generated for this study are available on request to the corresponding author.

## Author Contributions

SDu, SJ, and SDa developed the main idea. SDu performed all the measurements. SDu, JG, and KN performed the circuit simulations. CS performed the machine learning simulations. All authors discussed the results, agreed to the conclusions of the manuscript, and contributed to the writing of the manuscript.

## Conflict of Interest

The authors declare that the research was conducted in the absence of any commercial or financial relationships that could be construed as a potential conflict of interest.
